# Sensitivity of multispecies maximum sustainable yields to trends in the top (marine mammals) and bottom (primary production) compartments of the southern North Sea food-web

**DOI:** 10.1371/journal.pone.0210882

**Published:** 2019-01-28

**Authors:** Moritz Stäbler, Alexander Kempf, Sophie Smout, Axel Temming

**Affiliations:** 1 Leibniz Centre for Tropical Marine Research (ZMT), Bremen, Germany; 2 Institute for Hydrobiology and Fishery Science (IHF), University of Hamburg, Hamburg, Germany; 3 Thünen-Institute of Sea Fisheries,Bremerhaven, Germany; 4 Scottish Oceans Institute, East Sands, St Andrews, United Kingdom; University of New Haven, UNITED STATES

## Abstract

In marine ecosystems, maximum sustainable yield considerations are affected by any substantial changes that occur in the top and bottom compartments of the food-web. This study explores how the southern North Sea’s fisheries may need to adjust their fishing efforts to maintain optimum yields of sole, plaice, cod and brown shrimps under increased marine mammal populations and a reduced primary productivity. We constructed plausible scenarios of ongoing food-web changes using the results of Bayesian age-structured population models to estimate carrying capacities of harbour porpoises (*Phocoena phocoena*) and grey seals (*Halichoerus grypus*). Losses in primary productivity were predicted by lower trophic level ecosystem models. These scenarios were implemented in a food-web model of the southern North Sea. For each scenario, we sought mixed-fleet fishing efforts that would deliver maximum yields of sole, plaice, cod and brown shrimp combined. We also did so for a baseline run with unaltered mammal and primary production, and compared the differences in optimal fishing strategies, predicted yields, and states of the stocks between the scenarios. We found stocks and yields to be far more sensitive to changes in primary productivity than to increased marine mammal predation. The latter predominantly impacted cod, and even benefitted brown shrimps compared to the baseline run. Under 30% reduced primary productivity, fishing efforts had to be reduced by 50% to still provide maximum yields, whereas the marine mammal scenario induced no need to adjust the fishing regime. This draws attention to the potential gains of incorporating bottom-up processes into long-term management considerations, while marine mammal predation may be less of a concern, in particular for flatfish fisheries in the North Sea, and may even benefit shrimp trawlers because of reduced predation on shrimp from fish predators.

## Introduction

**Fig 1:**
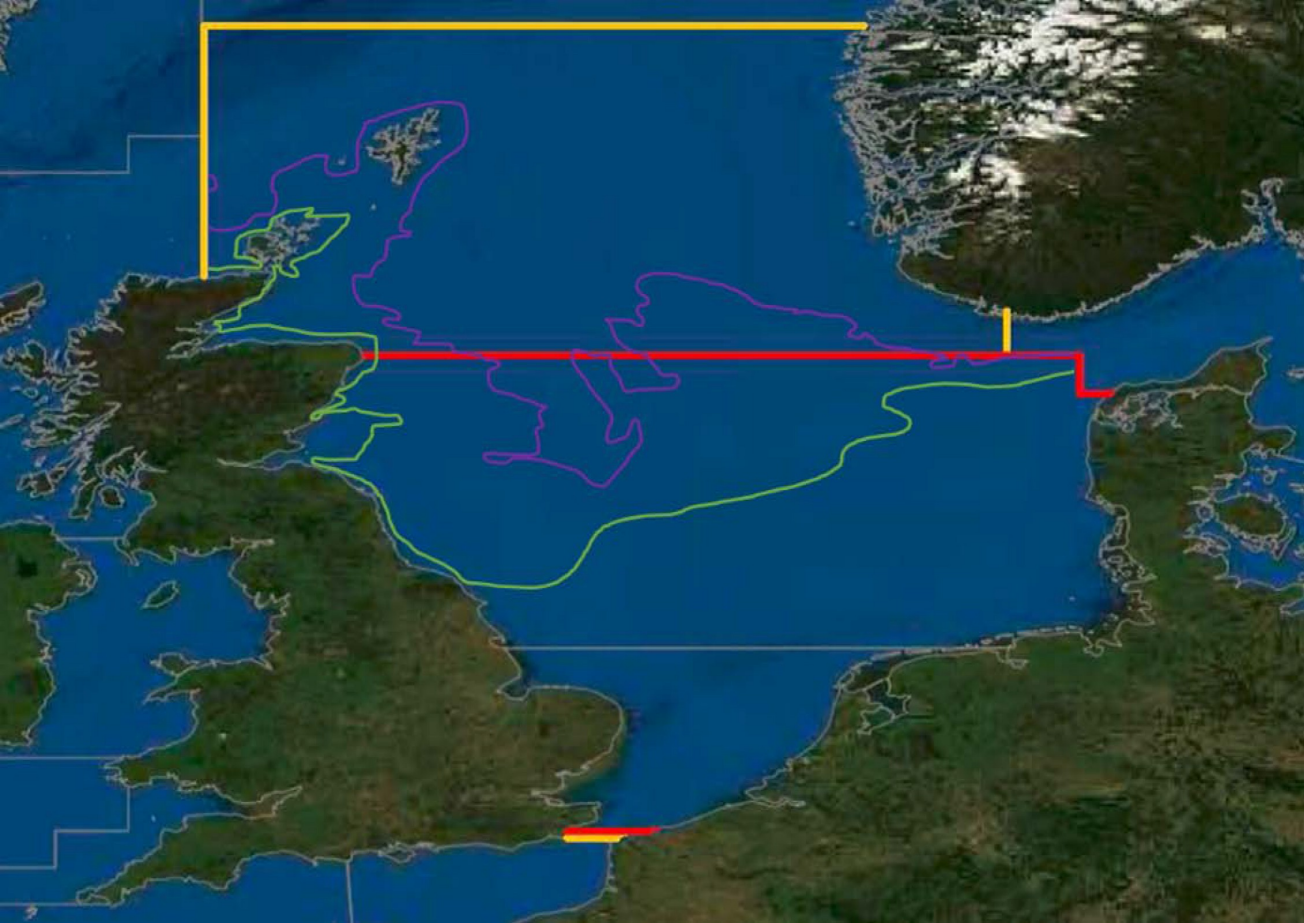
Map of the modelled area. As in [2]: statistical areas IVb and c (encircled red), within the total North Sea, statistical areas IVa, b, and c (encircled yellow) of the International Council for the Exploration of the Sea (ICES). 50 meters and 100 meters isobaths are sketched in light green, and purple, respectively. Adapted from http://gis.ices.dk/sf/index.html.

Managing fisheries for cod (*Gadus morhua*), plaice (*Pleuronectes platessa*), sole (*Solea solea*) and brown shrimp (*Crangon crangon*) in the southern North Sea (divisions IVb and IVc of the International Council for Exploration of the Sea, ICES; Fig 1) is a challenging enterprise, as the various target species are linked to each other through a complex food-web [1–3]. Also, one and the same species can be extracted by different gears with different consequences for other stocks and life stages and the environment [4–7]. Both multispecies and mixed-fleet effects have consequences when considering maximum sustainable yield (MSY) options for the area, questioning whether maximum yields of all single stocks can be achieved simultaneously [2, 6, 8]. Rather than single species MSYs, the goal in such cases should be the achievement of a multispecies MSY (msMSY), in which the trade-offs of fishing trophically or technically interlinked species are balanced such as to generate optimum aggregated outcomes to fishers and society [2, 3, 8].

But management of the southern North Sea’s living resources is not only confronted by trophic and technical interactions between fished stocks. The ecosystem is also subject to changes and trends in its system properties. Since the beginning of the 1980s, de-eutrophication measures led to reduced riverine discharges of inorganic phosphorous [9]. This decrease of nutrient availability can affect primary and secondary production [10, 11], a change in system productivity which bears the potential of cascading through the food-chain to affect exploited populations and fishing yields [10].

At the other end of the southern North Sea’s trophic spectrum, populations of marine mammals, namely harbour porpoise (*Phocoena phocoena*) and grey seals (*Halichoerus grypus*) have recovered from low densities in previous decades and may grow even further in future (c.f. [12, 13]; S1 File; and S2 File). Additional to the recovery of their total North Sea population, harbour porpoises appear to be moving their centre of distribution southwards into the south-eastern North Sea (SCANS I and II surveys). The enhanced marine mammal stocks can have direct negative effects on commercial species, if those contribute to the mammals’ diets [1]. However, this competition between fishers and marine mammals is by far not self-evident [14, 15]. For those stocks that have their predators removed by mammals, the effect can even be indirectly positive [1, 2, 16].

In a food-web model of the southern North Sea [2], we sought fishing effort levels for the three primal fleets of the area, demersal otter trawlers, purser and seiners (DEM), beam trawlers (BT) and brown shrimp trawlers (SHR), that would lead to a msMSY of cod, plaice, sole and brown shrimp. This effort regime and model setup would form the baseline scenario. We then subjected the modelled ecosystem to alternative scenarios and repeated the search for fishing efforts leading to msMSY. With the new msMSY found, we documented differences in biomass (B), catches (C), fishing mortality (F, where F = C/B) and revenue from landings per species. We compared each scenarios’ msMSY outcomes with the baseline scenario to quantify the sensitivity of msMSY to changes in modelled system properties and assumptions. The tested scenarios were:

1. Decrease in system productivity (e.g. through de-eutrophication measures)
2. Increase in the abundance of marine mammals, under
  - Ongoing southwards drift of the porpoise population
  - Halt of the southward migration of porpoises’ centre of distribution

For each such potential change of the modelled system, we investigated if it would lead to a need to adapt fishing strategies (as expressed by fishing effort and mortalities) to achieve msMSY and the consequences it would cause to yields, revenues, and stock biomasses.

## Methods

### A multispecies MSY for the southern North Sea flatfish, brown shrimp and cod fisheries

To establish estimates of fishing effort regimes which would lead to msMSY of cod, plaice, sole and brown shrimp, we used a time-dynamic food-web model of ICES divisions IVb and IVc [2, 17] (Fig 1). The model follows the Ecopath with Ecosim (EwE) food-web modelling approach and utilizes the dedicated software, version 6.4.11414.0 [18]. Parametrizing an EwE model typically starts off with setting up of an Ecopath model, the time-static snapshot representation of the ecosystem’s average state throughout a year. A set of linear equations covers the exchange of mass between the different biomass pools (or ‘functional groups’) of the model, where the flows into and out of any single group are characterized by the equation

$$B_{i}\cdot \left(\frac{P}{B_{i}}\right)=\sum_{j=1}^{n}B_{j}\cdot \left(\frac{Q}{B}\right){}_{j}\cdot DC_{ij}+Y_{i}+E_{i}+BA_{i}+\left(\frac{P}{B_{i}}\right)\cdot B_{i}(1-EE_{i})$$ Eq 1,

where Bi = biomass of functional group i; P/B = production per unit of biomass of the functional group i; (Q/B)j = consumption per unit of biomass of the predator j of biomass Bj; DCij = proportion of prey i in the diet of predator j; Yi = exports from the system as fishery catches; Ei = net migration; and EEi = ecotrophic efficiency of the functional group i. Energetic costs for the respective groups are described by Eq 2:

Consumption(Q)=Production(P)+respiration(R)+unassimilatedfood(U) Eq 2

Based on that snapshot representation of the ecosystem, Ecosim adds the dimension of time to the dynamics of the functional groups of the food-web. It simulates the development of the biomass of each functional groups as response to internal system structure (the underlying Ecopath) and external drivers, e.g. fishing or environmental changes, according

$$\frac{dBi}{dt}=gi\sum_{j}Qji-\sum_{j}Qij+Ii-(Mi+Fi+ei)Bi$$ Eq 3;

where dB_i_ is the growth of biomass of functional group i; g_i_ is its growth’s net efficiency, i.e. production/consumption; I_i_ is immigration rate; while e_i_ is emigration rate; M_i_ represents the non-predation natural mortality rate; and F_i_ is fishing mortality rate. Christensen and Walters [19] and Christensen and colleagues [18] expand further upon EwE’s compartments and assumptions beyond these base equations.

The EwE model designed for the southern North Sea covers 68 functional groups. As a characteristic of a food-web, or ecosystem model, these span from the very bottom (phytoplankton, benthic and pelagic microflora and invertebrates) to the top (marine mammals, sharks and seabirds) compartments of the represented ecosystem rather than commercial species (like multispecies models) or commercials with some top predators (minimum realistic models [20]). However, in the southern North Sea Ecopath model, fisheries’ target species were implemented in particular detail, including the representation in so called multi-stanza groups, with adults and juveniles modelled separately. Wherever possible, single species dynamics, including stock-recruitment dynamics, were tuned to single or multispecies stock assessment data [2]. The Ecopath model, and thus base year of the Ecosim simulations, is 1991, since the ICES ‘year of the stomach’ [21] provides uncompeted availability of fish diet data for that particular year. Fitting the Ecosim model to biomass, abundance, catch, fishing mortality and fishing effort data ranging 1991–2010 ascertained the best achievable plausibility of the model and its anticipated ability to predict future developments under changing external pressures. Stäbler and co-authors [2] provide further details about the model and its applications.

**Fig 2:**
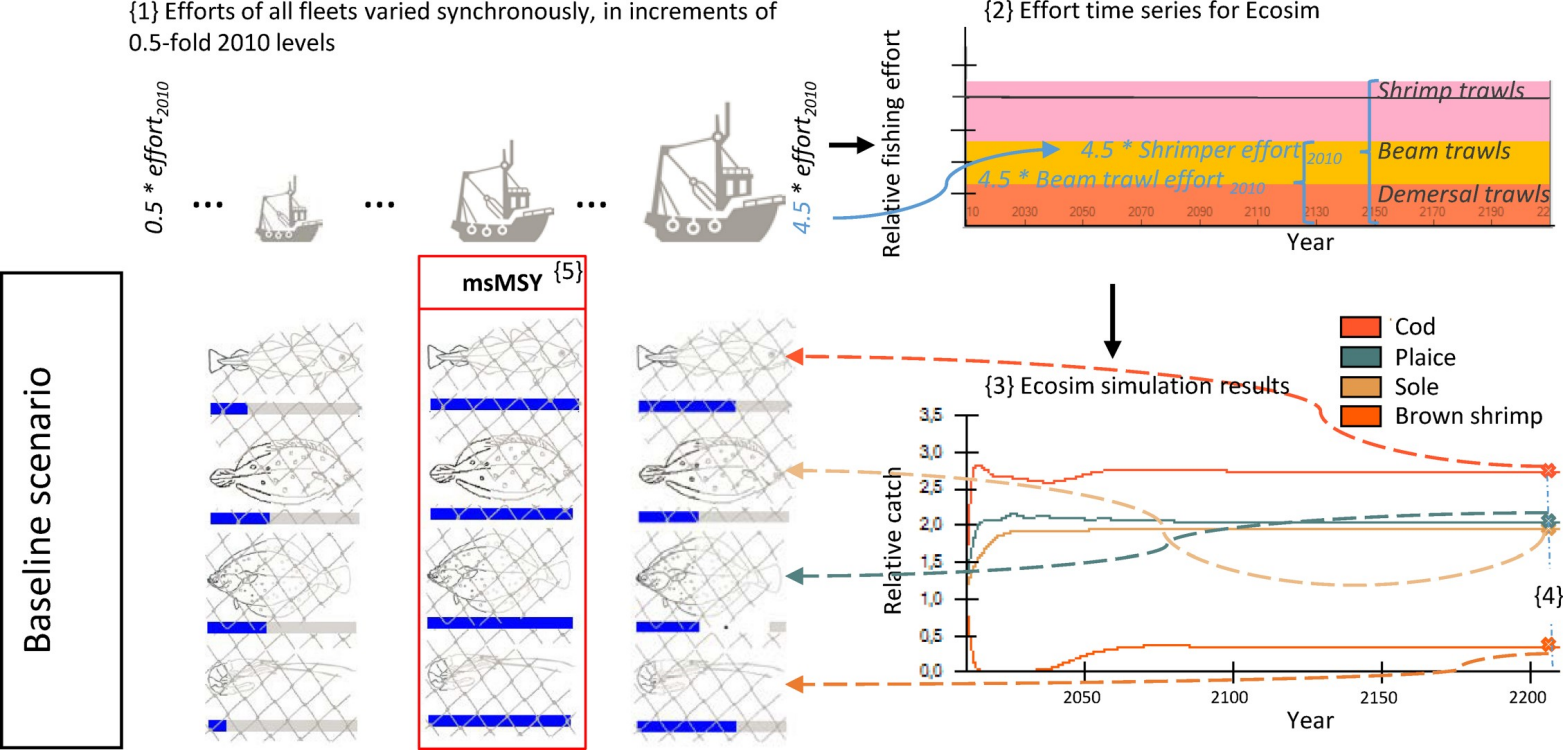
Visualization of the simulation design used to detect multispecies MSY (msMSY) in the baseline scenario. Sketched input and output parameters are hypothetical values for explanatory purpose only. They do not represent actual simulation results.

In search of a multispecies MSY for fisheries on plaice, sole, brown shrimp and cod, we altered the fishing efforts of the major fleets, DEM, BT and SHR, while keeping the effort ratios between the fleets stable, which means that if the effort of fleet *a* was increased by a factor x, the same factor was applied to efforts of the other fleets *b* and *c* ({1} in Fig 2). Our decision to screen for msMSY with stable ratios between the fleets is based on the concept of *relative stability*, which is a corner stone in EU fisheries management and ensures that each year a member country gets the same percentage of total allowable catch from a given species in a certain management area. In several countries (e.g., Germany) quotas are even distributed over vessels based on historic fishing rights. Therefore, a free distribution of quotas over fleets is not likely in the near future. We mimic this by keeping the relative share of each fleet constant and therefore just scale effort up and down without changing the distribution of effort (and thereby catches) between fleets.

The search for msMSY was conducted in a set of equilibrium simulations from 2010 (the end of the Ecosim hindcast which the model was fit to) onwards. Efforts were altered prior each such simulation run ({2} in Fig 2), and the system then run for 200 years, so it had long reached its equilibrium state ({3} in Fig 2). For the next-to-last year of these 200 years ({4} in Fig 2), catch, biomass and fishing mortality were recorded. Multispecies MSY was considered to be reached at the common level of increase, or decrease, of all three fleets combined, which would produce maximum total equilibrium yields from the four scope species combined ({5} in Fig 2). This definition of msMSY differs from the method employed in an earlier work by Stäbler and colleagues [2] by lumping together catches of all four species to form one indiscriminative catch pool to be maximized. We differentiated between yield in tonnes caught (msMSYt) and revenues from landings in Euros (msMSY€), for which we multiplied tonnes caught with 2014 off-vessel prices (German landings declarations, collected according to REGULATION (EC) 1224/2009 ON FISHERIES CONTROL; http://eur-lex.europa.eu/legal-content/EN/ALL/?uri=CELEX:32009R1224). In our search for maximum yields, only catches of the scope fleets were considered, while other fishing activities, such as gill netters or pelagic fleets, were left aside. We recorded B, F and catch in tonnes and revenues from landings (€) for cod, plaice, sole and brown shrimp at msMSY as outcome of our baseline scenarios ({5} in Fig 2).

In a first analysis, and an attempt to contrast msMSYt against msMSY€ with unaltered higher and lower TLs, we sought msMSYt, and msMSY€ at fishing effort increments of 0.1-fold the fishing efforts executed in 2010. The results of this analysis lead us to forgo the fine search grid of 0.1-fold 2010er efforts in the comparison between msMSYs attained under the different scenarios (c.f. Results).

**Fig 3:**
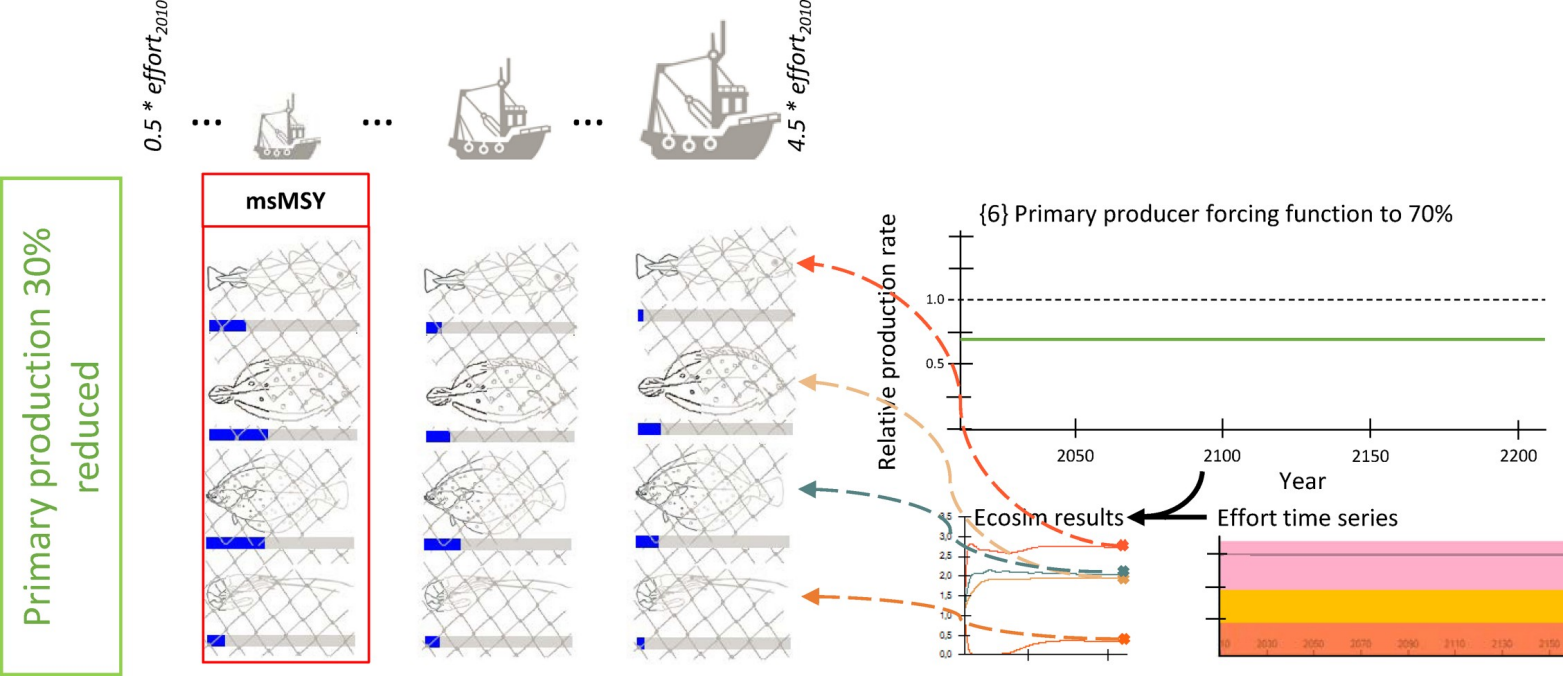
Visualization of the simulation design used to detect multispecies MSY (msMSY) under 30% decreased primary productivity. Sketched input and output parameters are hypothetical values for explanatory purpose only.

**Fig 4:**
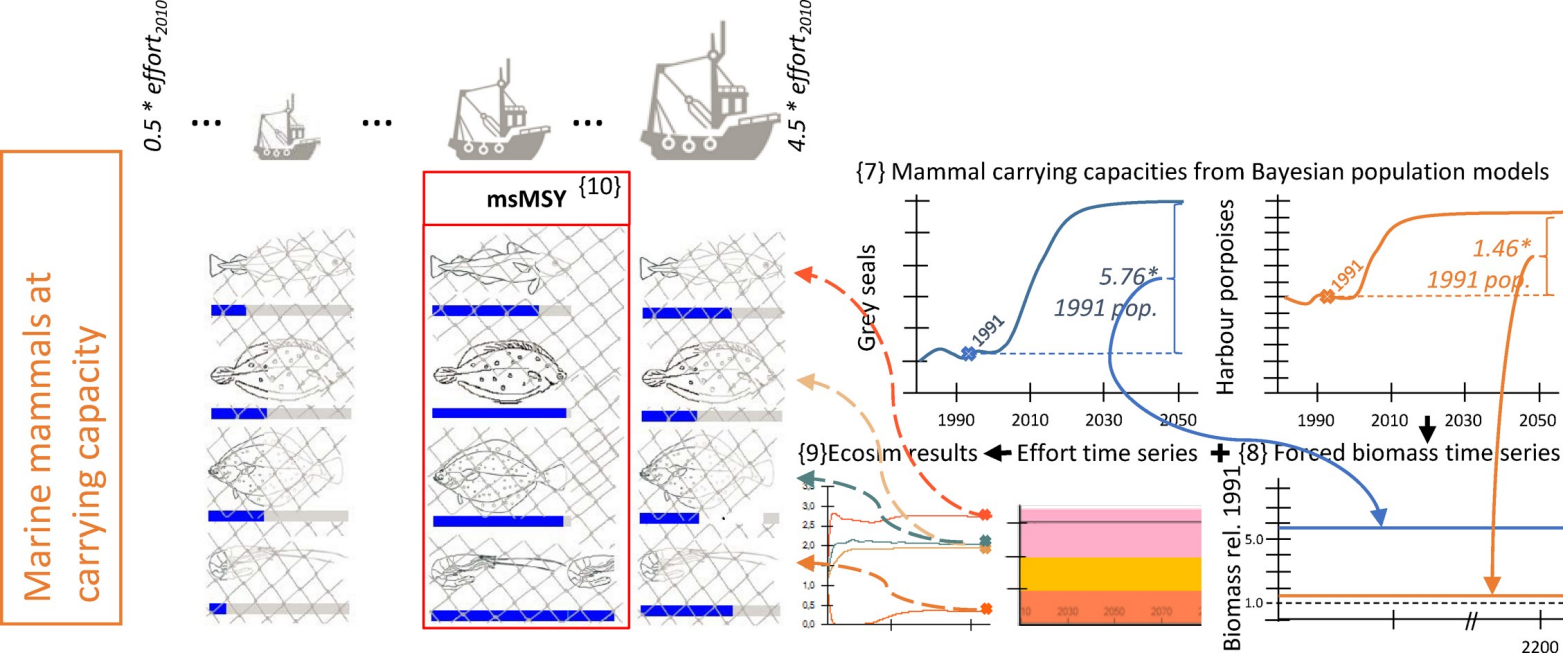
Visualization of the simulation design used to detect multispecies MSY (msMSY) with Grey seals and Harbour porpoises at predicted carrying capacities without ongoing southward migration of the porpoise population’s centre of distribution. Sketched input and output parameters are hypothetical values for explanatory purpose only.

**Fig 5:**
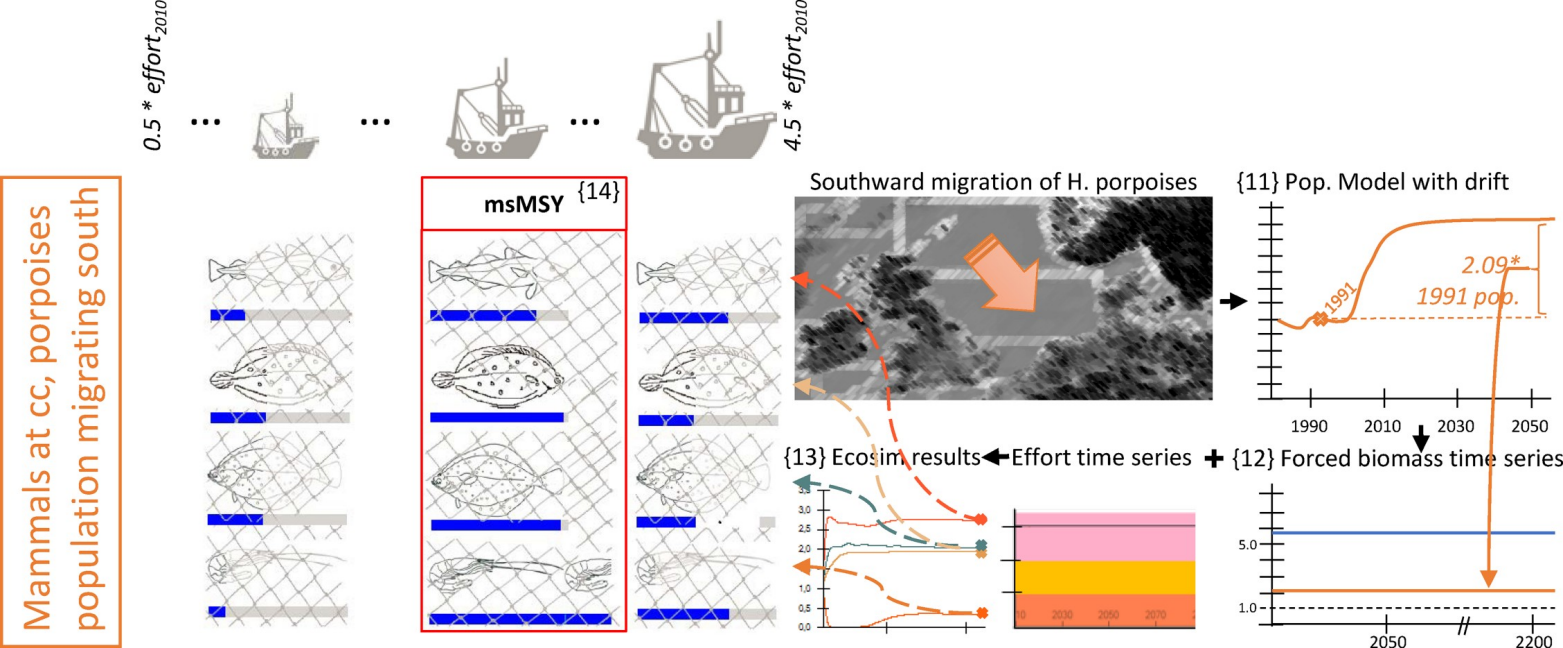
Visualization of the simulation design used to detect multispecies MSY (msMSY) with Grey seals and Harbour porpoises at predicted carrying capacities with ongoing southward migration of the porpoise population’s centre of distribution. Sketched input and output parameters are hypothetical values for explanatory purpose only.

The comparisons between scenarios (with, and without the changes in the food-web) were based on msMSY searches with fishing effort increments of 0.5-fold 2010er levels. We first established the baseline msMSY ({1} to {5} in Fig 2), and then repeated the search for msMSY as described above, but prior to that changed the properties of the modelled ecosystem as described below (Figs 3–5). In total, three alternative scenarios were tested: A decrease in system productivity (Fig 3), and increases in the abundance of marine mammals without (Fig 4), and with ongoing southwards drift of the harbour porpoise population (Fig 5).

### Decrease in system productivity

To overcome eutrophication phenomena, such as local anoxia [22] and changes in plankton species composition [10, 23], measures to reduce the nutrient loads into the catchment area of the southern North Sea were and are being applied [9]. The respective PARCOM 1988 convention foresaw a reduction of nutrient loads to 50% of the 1985 level until 2010 [24]. Besides their intended effect of counteracting phytoplankton blooms and associated processes like hypoxia and shifts in phyto- and zooplankton species composition that may cascade through the food-web [23, 25], cuts of the nutrient loads have the potential to affect fisheries’ yields and policies: By reducing the productivity and biomass of algae, which again serve as the basis of the marine food-web and eventually feed into exploited stocks, efforts to overcome eutrophication may lead to reduced stock productivities. To name an example, positive relationships between somatic growth or recruitment success, and nutrient loads were found for brown shrimp [26, 27] and plaice [28].

We evaluated how a decrease in net primary production (NPP, i.e. gross primary production (photosynthesis) minus respiration) of the maximum by -30% projected by Lenhart and colleagues [11] would cascade through the food-web and affect fishing yields under msMSY considerations. We therefore reduced primary productivity to 70% of its original value in our food-web model to create a scenario representing the decreased system productivity predicted by their study. The reduction in primary productivity was implemented as a *primary producer forcing function* in Ecosim [18], which reduced primary producers’ production rate to 70% of its original value for the entire simulated time period of 200 years ({6} in Fig 3), at the end of which (“in equilibrium”) catches and biomasses were recorded (analogue to {4} in Fig 2). To some extent, this approach is an example of an offline, one way model coupling exercise: Lenhart and co-authors [11] used an ensemble run of six different lower trophic level models (nutrients to zooplankton, with a physical model compartment) to generate predictions of primary production in the North Sea under a standard run and a 50% nutrient reduction scenarios. The across-model average maximum difference in NPP between the standard runs and the reduction scenarios (-30%, see section 4.2 in [11]) is their output we then build upon for the construction of our de-eutrophication scenario.

### Increase in the abundance of marine mammals

Both the populations of grey seals and harbour porpoises in the southern North Sea have increased through the last three decades. In the case of grey seals, this population growth is related to a recovery since the 1970s, when hunting and diseases had severely reduced the population (ICES *North Sea Ecosystem Overview* 2008; retreived from http://www.ices.dk/sites/pub/Publication%20Reports/Advice/2008/2008/6.1-6.2%20North%20Sea%20Ecosystem%20overview.pdf; 2016-JAN-16). We carried out projections based on Bayesian age-structured population models developed for assessment for grey seal population size from pup count and moult count data (see S1 File) which suggest that the current population is still far from its carrying capacity, but given current population model estimates, may grow to almost six fold its 1991 biomass ({7} in Fig 4). For our scenarios representing an increase in marine mammal abundance we therefore forced the biomass of the respective functional group ‘seals’ in our model to a level of 5.76 times its 1991 value. The group would then hold this biomass throughout the entire time period simulated when searching msMSY ({8} in Fig 4).

In the original fitted Ecosim model [2], the ‘*vulnerability*’, i.e. the availability of prey pools to seal predators [18], was estimated 1.0. This implies that the trophic flow from prey pools into the seal predator pool would be entirely bottom-up controlled, increases in the predator biomass would thus not result in a noticeably increased consumption of its prey. Inside Ecosim, a predator stock with *vulnerability* (v) close to 1 would, if its biomass e.g. doubled, drastically decrease its consumption per unit of biomass (Q/B) and hence quickly decline back to its original biomass [18]. For our analysis, we thus set the *vulnerability* of prey to seals to 2.0, which is the default value suggested by Christensen and co-authors [18] and typifies mixed control, i.e. neither bottom-up nor top-down processes [29] dominantly control the populations’ dynamics. With v = 2.0, seals would, if drastically increased, be able to double the predation mortality they cause to their prey. We changed the v of seals from 1.0 to 2.0 in the marine mammal scenarios only, not in the baseline or primary productivity scenarios. Practically, this meant valuing the comparability between the latter two higher than that between them and the mammal increase. We perceived this as the more parsimonious approach over the alternative of changing v to 2.0 for all three scenarios.

Harbour porpoises form the most abundant cetacean species of the southern North Sea. Between the SCANS surveys in 1995 and 2005, their centre of distribution has moved here from the northern part of the North Sea [30]. We implemented two different scenarios for our msMSY analysis, one in which the southward shift of porpoises that was observed between the SCANS I and SCANS II surveys would continue (Fig 5), and one in which the distribution shift was assumed to end (Fig 4). For both versions, we estimated the southern North Sea’s population’s carrying capacity using a designated population model that considers, amongst other factors, current bycatch rates of porpoises in fishing gears (cf. S2 File; {7} in Fig 4 and {11} in Fig 5). In the case of an ongoing southward migration of the population, the carrying capacity of harbour porpoises would reach 2.09-fold the biomass that the population held in 1991 ({11} in Fig 5). At a stopped drift this figure would be 1.46 ({7} in Fig 4). Such numbers can be considered plausible, given that Camphuysen [13] reported an average annual 41% increase in the number of sightings of harbour porpoises between 1989 and 2004, corresponding with a comparable increase in the number of reported strandings. The respective values were applied to the Ecosim functional group ‘toothed whales’ in our model: biomasses of porpoises were forced (as a *forced biomass time series* in Ecosim [18]) to values 2.09- ({12} in Fig 5 and 1.46-fold ({8} in Fig 4) times the biomass parameterized for 1991 in the Ecopath models. These biomass forcing functions were applied for the entire 200 years time span, at the end of which catches and biomasses were recorded to represent equilibrium state and yields ({9} in Fig 4 and {13} in Fig 5). As in the previous scenarios, the fishing effort regime (as a synchronous level of increase, or decrease, of all three scope fleets’ efforts) leading to the highest bulked catches was deemed the scenarios’ msMSY ({10} in Fig 4 and {14} in Fig 5).

Combined with the above described increase in seal biomass, harbour porpoise increases to the two different carrying capacities formed our two marine mammal scenarios to test the sensitivity of msMSY to changes in these species: one in which grey seals and harbour porpoises would reach their anticipated carrying capacities, and one in which this would be the case, but porpoises would additionally keep up their southward migration. Both scenarios are speculative because they project forward based on previous trends. For the seals, the best projection from the population model available was used, but it should be noted that the carrying capacity of a system for a population that is growing from lower numbers is numerically difficult to estimate because the population has not yet reached its asymptote. For the porpoises, two different assumptions about future distributional trends were utilised without a good understanding of the drivers that caused the 1995/2005 re-distribution. However, these assumptions did allow us to achieve our aim to explore the behaviour of the ecosystem under substantial increased predation pressure from the marine mammal species and to compare this with the effects of bottom-up processes.

## Results

**Fig 6:**
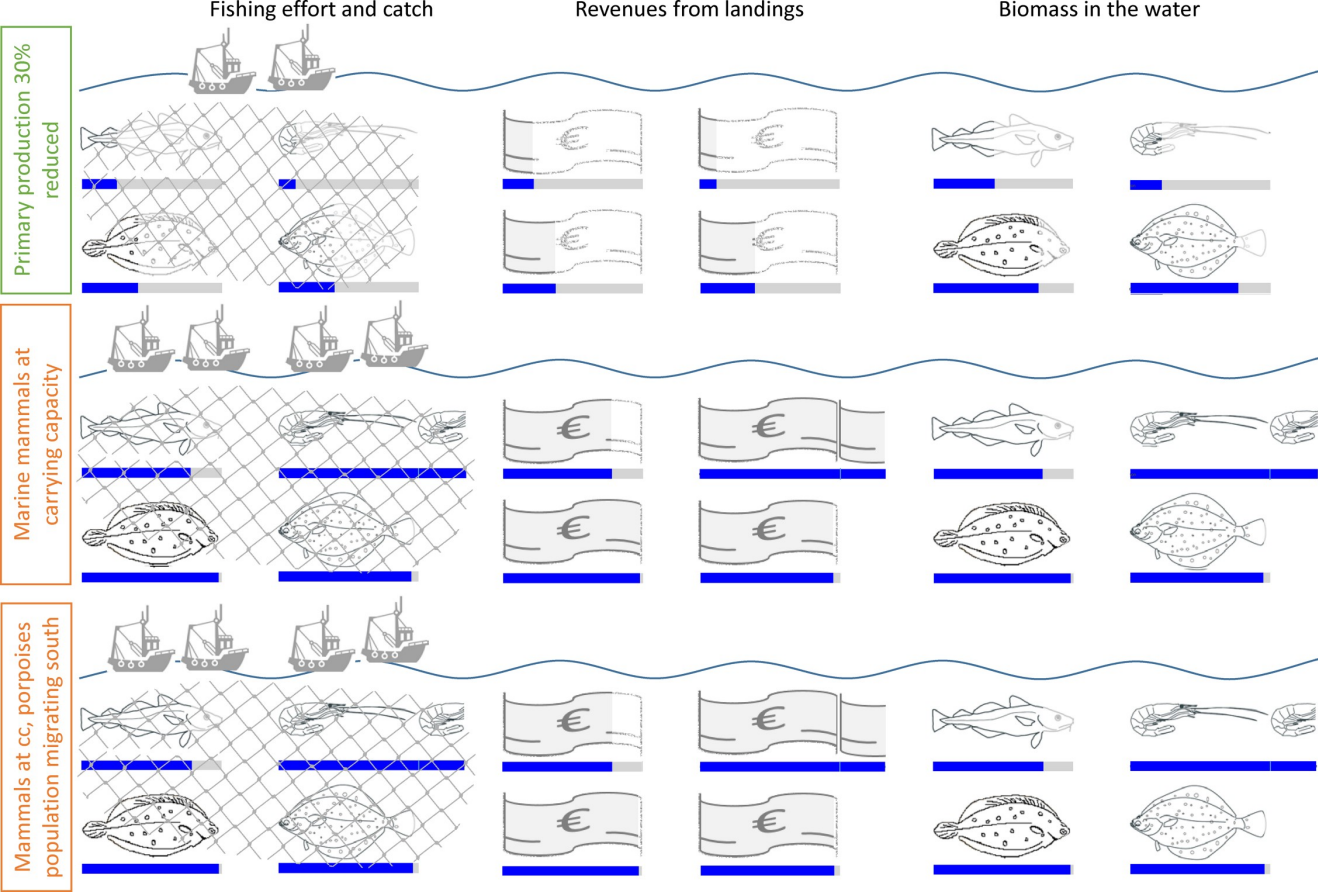
Relative fishing efforts leading to msMSY under changed ecosystem properties (30% reduced primary productivity, and marine mammal populations at carrying capacity); with respective catches, revenues from landings, and stock biomasses of cod, brown shrimp, plaice and sole (clockwise, starting top left) relative to baseline scenario. Icons partially adopted from [31].

In search of fishing strategies leading to msMSY in the different scenarios (baseline, primary productivity de-, and marine mammal increases), fishing efforts were changed in steps of 0.5-fold those efforts executed in 2010 (c.f. first section of *Methods*). After finding msMSY for the baseline scenario, we contrasted the results with the scenario of projected future marine mammal populations. Our results suggest that increases in the abundance of marine mammals do not lead to a need to reconsider the fishing strategy leading to msMSY. At a 0.5-fold search grid (c.f. first section of *Methods*), there are no differences in effort and F levels between the two marine mammals scenarios and the baseline (Table 1). Increases in the populations of harbour porpoises and grey seals considerably affect cod catches and biomasses (and more so, as expected, for the scenario with pertained southwards drift of the porpoise population), while they have a much more limited effect on flatfish (Fig 6, Tables 2 and 3). As suggested by Temming and Hufnagl [1], our study also shows that seal predation on cod relieves brown shrimp from their key predator, leading to higher stock biomasses and catches of shrimp (Fig 6, Table 3). The only subtle differences between the scenario with and the one without ongoing southward shift of harbour porpoises illustrates the dominant impact of seals on fished stocks compared to the cetaceans.

**Table 1:** Fishing mortalities (F) of mature cod, plaice, sole, and brown shrimp, and fishing efforts at multispecies MSY under different scenarios.

Scenario	Effort relative to baseline	F Cod	F Plaice	F Sole	F Brown shrimps
**Baseline**	/	0.45	0.40	0.33	2.46
**PP 70%**	0.5	0.26	0.21	0.18	1.23
**Mammals with drift**	1	0.45	0.40	0.33	2.46
**Mammals without drift**	1	0.45	0.40	0.33	2.46

**Table 2:** Equilibrium spawning stock biomasses at multispecies MSY under different scenarios relative to baseline multispecies MSY.

Scenario	SSB Cod	SSB Plaice	SSB Sole	B Brown shrimps
**PP 70%**	44%	77%	75%	23%
**Mammals with drift**	78%	95%	98%	134%
**Mammals without drift**	79%	96%	98%	133%

**Table 3:** Catch in tonnes at respective values of multispecies MSY relative to baseline scenario's multispecies MSY.

Scenario	C_t_ Cod	C_t_ Plaice	C_t_ Sole	C_t_ Brown shrimps
**PP 70%**	25%	40%	40%	12%
**Mammals with drift**	78%	95%	98%	134%
**Mammals without drift**	79%	96%	98%	133%

A cut in system productivity, as modelled through the 30% decreased primary productivity, has severe consequences on the food-web and its fisheries. Efforts and thus Fs (given that the relationship between F and effort is assumed linear) have to be reduced to half the levels they would be at msMSY with a primary production as is (Fig 6, Table 1). This counts for both msMSYt, i.e. the fishery optimized to catch a maximum total tonnage of the four scope species combined, as well as msMSY€, the strategy leading to the largest overall revenue from landings of the four species. Brown shrimps are struck the hardest, having their biomass reduced to less than a fourth of the baseline msMSY value (Fig 6, Table 2). With brown shrimp being a prime prey organism of cod in the model, it comes as no surprise that the latter suffers severely from reduced system productivity cascading through the modelled food-web. Its biomass declined below half of what was found at the baseline msMSY (Fig 6, Table 2), despite considerably lower fishing pressures (Table 1). Flatfish SSB are the least affected by a reduced primary productivity, but still lose a fourth of their biomass compared to the baseline scenario (Fig 6, Table 2). Their catches and revenues from landings are more affected and drop to around 40% (Fig 6, Tables 3 and 4). Catches of cod are depleted to a fourth, and shrimp fishery is affected the strongest with its yields down to a tenth of what was produced as the baseline msMSY.

**Table 4:** Revenues from landings at respective values of multispecies MSY relative to baseline scenario's multispecies MSY.

Scenario	C_€_ Cod	C_€_ Plaice	C_€_ Sole	C_€_ Brown shrimps
**PP 70%**	22%	39%	38%	12%
**Mammals with drift**	78%	95%	98%	133%
**Mammals without drift**	79%	96%	98%	133%

At the 0.5-fold effort search grid we applied for the msMSY search under the four scenarios, differences between msMSYt and msMSY€ became indistinct and both fishing strategies were alike. At a finer search grid, differences, if also minor ones, would probably have occurred in all scenarios. To demonstrate that, we applied a finer search for the baseline scenario, and found efforts and fishing mortalities leading to msMSYt to be around 10% higher than those leading to msMSY€. This is for the contradictory biological and economic dynamics of plaice and sole: While plaice is the more solid of the two stocks in the southern North Sea, it also reaches only about 16% of the market price of sole. A fishing strategy opting for maximum yields in tonnes combined will thus plea for (slightly) higher efforts, resulting in higher catches of plaice but overfishing of sole, while the opposite will be the case for strategies aiming for high revenues from landings.

## Discussion

This study explores the sensitivity of msMSY fishing strategies and yields to projected ecological changes in a food-web model of the southern North Sea. It shows in which cases fishing pressures have to be adapted in response to potential future ecosystem regimes to produce maximum catches and revenues, and how yields and spawning stock biomasses may react. All potential environmental changes tested here have negative effects on the yields of the three fish species sole, plaice, and cod. Generally, plaice catches are most robust, followed by sole. Brown shrimp catches suffer from cuts in system productivity, but benefit from cod stock reductions through marine mammals. Of the scenarios tested, losses in primary productivity pose the most severe challenges to all three fisheries, beam, otter and brown shrimp trawlers, while the predicted increases in marine mammals consistently raise the least concerns.

The finding that fished stocks’ productivities would decline with primary productivity (representing nutrient reduction) in the model is well in line with results reported from empirical studies. As such, Rijnsdorp and Leeuwen [28] found hints that juvenile plaice in nearshore areas of the North Sea may grow worse under lower nutrient loads, alike to findings for sole [27] and brown shrimps [26]. For freshwater lakes, de-eutrophication measures (i.e. reductions of nutrient input) have been demonstrated to impair fisheries production for some cases [32, 33], whereas other studies found no effect [34]. *In vivo*, stock and yield declines under the de-eutrophication might be counteracted by other, positive effects of reduced nutrient loads, e.g. arising from a lower likelihood of bottom oxygen deficiencies. These oxygen depletions can occur if excess production of phytoplankton organisms leads to them sinking to the sea floor before they can get grazed in the water column. On the bottom, this excess organic matter is consumed through oxygen intensive microbial processes, which can lead to local oxygen depletion. Hypoxia, also termed oxygen deficiency, which regularly occurs in the southern North Sea [35], makes brown shrimp metabolism less efficient [36], and impairs the egg development [37] and year class strength [38] of cod in the Baltic Sea. By lowering the probability of such events, reduced nutrient loads can actually enhance resource productivity.

Besides decreasing the likelihood of hypoxia, further effects of de-eutrophication are rather complex to predict. This refers to nutrient-induced changes in the species composition of phyto- and zooplanktonic communities and those species’ respective attractiveness and availabilities (or *vulnerabilities*) to predators [10, 23]. The plankton groups are only coarsely represented in our Ecosim model. Zooplankton is constituted of three functional groups in the model, of which copepods form a single one. Hence, with regard to copepods, our model’s transfer of changes in primary productivity (in our case derived from external models; [11]) to higher trophic levels via zooplankton should be considered a simplified representation of a considerably more complex pathway, which may lead to us overestimating the efficiency of transversion of primary production into stock productivities. For cod, copepod species composition, rather than pure abundance, has been shown to affect stock productivity [23, 39]. That process would not be represented by our model. Neither resolved were species- respectively size-specific preferences or even non-edibility of phytoplankton by zooplankton. Lower nutrient availability leads to lower phytoplankton cell sizes. These smaller cells are easier to ingest by zooplankton larval stages, compared to the large cell sizes occurring during high nutrient conditions. Reducing nutrient loads could hence neutralize the potential decoupling of zooplankton from phytoplankton growth under eutrophication [40]. Copepods’ longer generation cycles compared to phytoplankton may make them incapable of skimming off boosted primary production in the North Sea [41]. Smaller, lighter cells growing during low-nutrient phases are less well armoured with calciferous or silicate shells, and sink slower, thereby making the phytoplankton biomass more available (*vulnerable*) to zooplankton in enhanced nutrient conditions [40]. Also microzooplankton (heterotrophic protozoans), being amongst the most important consumers in pelagic food-webs (and hence food for fish), prefers small celled dinoflagellates, cryptophytes and green algae over the often dominant diatoms [42]. A 30% reduction in primary production, as in our scenario, does thus not necessarily lead to 30% less food available for zooplankton, or via that fished stocks, *in vivo*.

While alternative, more detailed parameterizations of the model’s pelagic lower trophic level compartment would chiefly reduce the uncertainty associated to the simulation results of cod, they bear less relevance for flatfish, or brown shrimps, which have a more benthic diet. The negative trophic effect of low nutrient loads on the productivity of sole and plaice [27, 28] primarily arises from a lower benthic production under non-eutrophication [43–45]. Total benthos production can, even alongside a change of species composition, be fuelled through phytoplankton both edible and non-edible by intermediate trophic levels, given that the latter would end up as detritus and feed into the benthos compartment that way [46].

As stated, the potential positive effects of de-eutrophication and the complexity implied by the diverse phyto- and zooplankton communities and their feeding interactions were not included in the simulation modelling of this study. Given this, combined with the fact that all production in the model depends on primary production, phytoplankton decline was bound to impair predicted stock productivities and yields in the model. That said, it is noteworthy that the resulting loss in biomasses, yields and revenues is generally disproportionately higher than the implemented algae reduction, and that it cascades through the entire food-web to affect e.g. cod pretty much undamped. The response of flatfish is less severe, which probably relates to their more flexible diet [2, 46]. The plausibility of these modelled results could be underpinned by studying the past correlations of stocks’ productivities and nutrient loads. In a study which relates coastal nutrient loads with spatial distribution rather than stock productivity, Støttrup and colleagues [47] found indications that declines in coastal nitrogen loads have driven juvenile plaice away from the shore into deeper waters of the North Sea since the early 1990s. Colijn and co-authors [10] reviewed trends in the biomasses and productivities of plankton, benthos, fish and shrimps, and related these changes to nutrient enrichment. However, given the multitude of drivers affecting stock biomass and productivity and those drivers’ likely multicollinearities, they found the causes hard to disentangle. Until this has been successfully performed, our study presents a meaningful what-if simulation.

Considering the predicted consequences of marine mammal upsurge, even the strongest assumptions (including an almost six-fold increase of seal biomass) lead to disproportionately lower responses of biomasses and yields, in contrast to the amplified, overproportioned effect of the 30% reduced primary production. Should this appear unexpected at first, that expectation gets entirely reversed when looking at absolute changes in biomass or production in tonnes per annum: The standing stock of phytoplankton alone is 300 times higher than that of all marine mammals combined, whereas the annual total primary production (P/B*B = P) is six orders of magnitude higher than that of marine mammals in the Ecopath 1991 base model [46].

To launch our marine mammal scenarios, we set the *vulnerability* (v) of seals to 2.0 instead of the originally fitted 1.0. The EwE user guide [18] caution that the decision for the default value 2.0 is as valid as the decision for any other value, and the extent to which an increase of seal biomass affects its prey is a directly predictable function of that input parameter v. However, whether we choose v = 1.0 or v = 2.0 does not affect FmsMSY and efforts leading to msMSY, but only BmsMSY and CmsMSY (results not shown).

In our attempt to predict a carrying capacity of seals (c.f. third section in *Methods*), we only included grey seals in the analysis, but left harbour seals (*Phoca vitulina*) aside. This simplification is primarily due to the unavailability of an appropriate population model, and we consider it to be of minor importance in estimating potential future seal predation, as both populations appear to grow (ICES *North Sea Ecosystem Overview* 2008) and since grey seals contribute the major share of total seals’ biomass [48]. Another simplification is the spatial inexplicitness of the Ecosim model, while in fact seal predation may be a localized phenomenon [1], with both species returning to haul-out on land at regular intervals ([49]; ICES *North Sea Ecosystem Overview* 2008). While there should be considerable interest in the more detailed, spatially explicit modelling of seals and prey within an ecosystem framework, for this study, it is assumed that marine mammal predation is, if not spatially uniform, at least an omnipresent phenomenon in the southern North Sea. As such, grey seals can be seen far offshore (ICES *North Sea Ecosystem Overview* 2008), and harbour porpoises are sighted in high densities up to 300km off the nearest coast (SCANS I and SCANS II surveys). Still, the level of marine mammal predation we used in our scenarios can be considered to lie at the upper margin of expectable future developments. This is particularly the case for the southward migration scenario, given that results of the now available SCANS III survey (https://synergy.st-andrews.ac.uk/scans3/files/2017/05/SCANS-III-design-based-estimates-2017-05-12-final-revised.pdf) suggest that the number of porpoises in the southern North Sea might not have changed all that much.

Our findings of the fisheries on all four scope species being affected by changes in the upper and lower trophic levels calls for the consideration of the food-web (and ecosystem) that fisheries operate in, when attempting to manage them well. Seal and porpoise predation are not addressed in ICES’ stock assessments and management considerations of sole and plaice in the North Sea [50]. Very different so for cod: seal abundances and diet data are considered crucial in estimating the North Sea cod stock’s predation mortality [50], e.g. in the stochastic multispecies model SMS [50, 51]. Since currently no input nor output management scheme applies for brown shrimp in the North Sea, the issue of potential indirect effects between shrimps and mammal populations is not addressed on an ICES advisory level, but it was described by Temming and Hufnagl [1]. Effects of nutrient loads upon stock productivities are generally not referred to in the stock assessments reports of plaice and cod [50, 52], but the potential positive relationship between riverine phosphate discharge and the growth of sole [27] was mentioned under ‘ecosystem aspects’ in their 2014 assessment [52]. That is no surprise, given that, while impacts of selected upper trophic level predators on target stocks can be addressed by some multispecies models (e.g. SMS; see ICES *Working Group on Multispecies Assessment Methods* [51]), explicit implementations of lower trophic level dynamics and their direct and indirect interactions with target species are the unique feature of food-web models (or designated minimum realistic models; c.f. [20]) and cannot be included into the single species assessment models which dominantly produce ICES’ advice. That, however, does not impair single species models’ capacity to produce tactic advice, given that changes in lower trophic levels would generally happen throughout long time scales. It only affects their ability to provide possible explanations of where such long term changes in stock productivity could stem from. The larger set of explanatory parameters in food-web models, of course, comes for the price of walking the tightrope between overparametrization and boundless uncertainties [2, 20]. Thus, as any model, particularly data intensive ones like our Ecosim model, which is rich in input parameters and biomass pools, it inherits the uncertainties of its data sources [2, 8] and can be biased through the potentially flawed assumption of stable patterns in any of the input parameters, such as e.g. dietary preferences. The scenario outcomes reported here could look considerably different were the model parametrized even slightly different. Monte Carlo approaches or ensemble runs (e.g. using *Rpath*, the R version of the Ecopath with Ecosim model, see https://github.com/slucey/RpathDev) could be ways to quantify the uncertainties around model results in future studies.

Additional limitations arise from the application of the different scenarios. As such, our primary productivity setup is tangled with issues in any of the lower trophic level model runs synthesized by Lenhart and co-authors (c.f. second section in *Methods*). Also, the -30% primary productivity there applies for certain areas only, while we interpreted it as a whole area average in the case of de-eutrophication. This deems our productivity reduction scenario a rather extreme test of the system. Many of the de-eutrophication measures mentioned in the scenario modelling of Lenhart and colleagues [11] have already been undertaken, such that it cannot be taken for granted that the decrease in primary productivity we hypothesize would prevail.

## Conclusion

Our results indicate that exploitation intensities, i.e. fishing mortalities and efforts, leading to msMSY may be robust to changes in marine mammal predation but very sensitive to changes in system productivity, which would require us to reconsider fishing strategies when opting for maximized yields in weight and revenue. In essence, when the system was placed under stress, the level of exploitation had to be reduced in order to optimize yields. These results illustrate the benefits of fishing at the lower edge of MSY ranges, i.e. with fishing pressures that, while still providing acceptable yields, lie below those associated with absolute maximum yields [2, 3, 53, 54]. Regarding fishing intensities that theoretically provide absolute optimum yields as a limit that is to be avoided rather than a target may provide not only conservation safeguards, but also increase the robustness of aspired yields.

## Supporting information

S1 File Grey seal projections. (DOCX)

S2 File Harbour porpoise model. (DOCX)
