# Supplementary material for: Sensitivity of multispecies maximum sustainable yields to trends in the top (marine mammals) and bottom (primary production) compartments of the southern North Sea food-web
Source: PLoS One. 2019 Jan 28;14(1):e0210882. doi: 10.1371/journal.pone.0210882 (PMC6349316; doi:10.1371/journal.pone.0210882)
Supplement: S1 File — (DOCX) [file pone.0210882.s001.docx]

Supporting information 1: Grey seal projections

Sensitivity of multispecies maximum sustainable yields to trends in the top (marine mammals) and bottom (primary production) compartments of the southern North Sea food-web

*Moritz Stäbler^1,2^, Alexander Kempf^3^, Sophie Smout^4^ and Axel Temming^2^*

*^1^ Leibniz Centre for Tropical Marine Research (ZMT) – Fahrenheitstraße 6 – 28359 Bremen – Germany*

*^2^ Institute for Hydrobiology and Fishery Science (IHF), University of Hamburg – Olbersweg 24 – 22767 Hamburg – Germany*

*^3^* Thünen-Institute of Sea Fisheries – Herwigstraße 31 – 27572 Bremerhaven – Germany

*^4^ Scottish Oceans Institute – East Sands – St Andrews, KY16 8LB – United Kingdom*

# S1: Grey seal projections

## Introduction

This document describes the approach used to predict grey seal numbers and biomass in the southern part of the North Sea for use in the MYFISH project. The model structure and parameters are based on a Bayesian age-structured population model described by Thomas [1]. The model divides the UK grey seal breeding population into several areas of which the whole-North-Sea, excluding Orkney, is one. Model selection suggests that these breeding populations are separate, and the model assumes each of them is internally well-mixed without further spatial structure. We focus on the model outputs representing the grey seal population which breeds in the main part of the North Sea, which is considered a reasonable approximation to the population using the southern region of ICES area IV.

Future scenarios may involve different assumptions about the levels of fishing in fisheries that may result in seal bycatch, and/or the nature of density dependence in the seal population.

## Methods

- The population model was fitted to aerial survey pup count data, and summer count data for adult grey seals [1]. Pup count data are available for all years in the time series, but only one count survey for adults was completed. The model was fitted using informative priors (for survival and fecundity) based on long-term observational studies and on the analysis of historical shot samples [2]. Density dependence in this model acts though first-year pup survival, with low survival rates currently predicted for some UK populations (though not that in the North Sea which does not yet appear to be approaching carrying capacity).
- In order to interpolate/extrapolate the NS population, a simulation is provided using EXCEL based on the age-structured population model fitted for the North Sea area. To initialise the model, following the method of Thomas [1], the initial population structure is assumed. It is derived from a Leslie matrix model with values of survival and fecundity corresponding to a low-density population (i.e. showing very little effect of density dependence).
- To convert from numbers-at-age to biomass, I assumed the following masses–at-age in kg [3].

S1.1 Table: Mass-at-age conversion to convert numbers-at-age to biomasses.


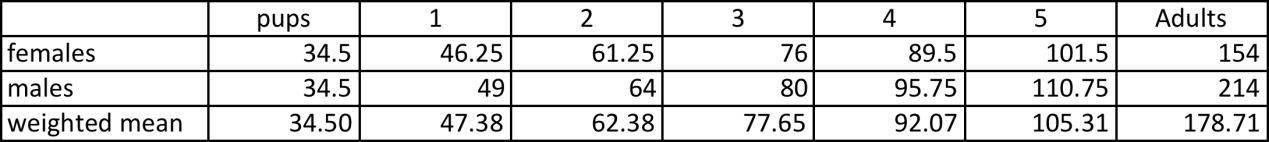


### Running the model to generate predictions

The baseline model has been run forward in time for 50 years from the present. Different scenarios that might be explored could include:

#### Adjust carrying capacity

Because the North Sea grey seal population does not currently appear to be close to carrying capacity, estimates of its carrying capacity based on current time series data sets are subject to considerable uncertainty. The effects of this uncertainty on the population projections can be investigated by altering the parameter chi, currently set at 14400. Plausible values to try, based on the posterior distribution of the parameter χ would be 12000, 16000 [1].

#### Include bycatch explicitly where future fishing effort intensifies

Currently, the grey seal population model does not explicitly include grey seal bycatch, because this is not thought to be an important driver for grey seal populations in the North Sea. Bycatch mortality that has been operating over the time represented in the data set will have been ‘absorbed’ into natural mortality during the model fitting process.

The effect of additional removals due to alternative, more intensive bycatch scenarios could be included using a simple subtraction representing ‘additional’ annual bycatch (see sheet 2 “remove adult females” for one example, in which ‘extra’ bycatch has been implemented from 2013 onwards).

The effect of bycatch on the population trajectory will depend on the impact on different age classes: removals of adult females will cause the greatest change in the population trajectory, while removals of juveniles will have less effect. In general with marine mammals, it is often the case that it is the naïve younger animals that experience greater mortality in interactions with fishing gear. For the sake of developing a speculative scenario it might be reasonable to allocate most of the bycatch to juvenile animals but we must be cautious in interpreting results. Because of low sample sizes in records of bycatch, very little data is available indicating the true relative vulnerability of different grey seal age classes to bycatch.

If a future scenario with increased effort in midwater trawling or in set net fishing is to be explored, the rates in the following table may be of use. Conservatively, it might be appropriate to assume the impact of all seal bycatch focussed on grey seals (for this assessment). Note that rates are not (within the range of available data) significantly affected by the grey seal population size.

S1.2 Table: Fisheries catch rates.

|  | *General gear type* | *Observations* | |  | *Catch rate per 1000 days at sea* | |
| --- | --- | --- | --- | --- | --- | --- |
|  |  | *No of hauls observed* | *No of bycaught individuals* | *Expected No of hauls per day* | *Mean* | *95% UCL* |
| Seal (2 spp). | All midwater trawl | 205 | 50 | 1.2 | 292.68 | [370.31] |
|  | All set nets | 6099 | 84 | 3.2 | 44.07 | 54.48 |

## Caveats

1. The numbers of observed bycaught animals are low and therefore this is noisy data, resulting in uncertain catch rate estimates. It is not possible to estimate the age-structure of by-caught animals and thus difficult to estimate any difference in vulnerability between age classes. It is possible that the overall calculated bycatch rates are biased low (Northridge pers com).
2. The population model assumes that UK populations of grey seals using the Orkneys, North Sea, and West coast are entirely separate. For breeding, this may be approximately true. However in practice during the foraging season there can be substantial movements between areas, and it is very likely that the impact of high levels of bycatch in one area would be “shared” by animals that breed outside this area. In the case of the North Sea, large numbers of animals breeding in Orkney are known to use the North Sea for foraging.

## References

1. Thomas L. Estimating the size of the UK grey seal population between 1984 and 2014 using established and draft revised priors. 2013 SCOS Briefing Paper.

2. Lonergan M. Detecting Density Dependence in Recovering Seal Population is Difficult: A Response to Svensson et al. (2011). Ambio. 2012;41(2):219-20. doi: 10.1007/s13280-011-0180-2. PubMed PMID: WOS:000303464700013.

3. Sparling CE. Causes and consequences of variation in the energy expenditure in grey seals (Halichoerus grypus): University of St Andrews; 2003.
