# Supplementary material for: Sensitivity of multispecies maximum sustainable yields to trends in the top (marine mammals) and bottom (primary production) compartments of the southern North Sea food-web
Source: PLoS One. 2019 Jan 28;14(1):e0210882. doi: 10.1371/journal.pone.0210882 (PMC6349316; doi:10.1371/journal.pone.0210882)
Supplement: S2 File — (DOCX) [file pone.0210882.s002.docx]

Supporting information 2: Harbour porpoise model

Sensitivity of multispecies maximum sustainable yields to trends in the top (marine mammals) and bottom (primary production) compartments of the southern North Sea food-web

*Moritz Stäbler^1,2^, Alexander Kempf^3^, Sophie Smout^4^ and Axel Temming^2^*

*^1^ Leibniz Centre for Tropical Marine Research (ZMT) – Fahrenheitstraße 6 – 28359 Bremen – Germany*

*^2^ Institute for Hydrobiology and Fishery Science (IHF), University of Hamburg – Olbersweg 24 – 22767 Hamburg – Germany*

*^3^* Thünen-Institute of Sea Fisheries – Herwigstraße 31 – 27572 Bremerhaven – Germany

*^4^ Scottish Oceans Institute – East Sands – St Andrews, KY16 8LB – United Kingdom*

# S2: Harbour porpoise model

## Introduction

This document describes the approach used to predict harbour porpoise numbers and biomass in the southern part of the North Sea for use in the MYFISH project. The methodology is based on a Bayesian age-structured population model developed by Winship [1] which includes bycatch as a source of mortality for the animals in addition to natural background mortality. The model used has the whole-North-Sea as one mixed population. Therefore to allocate numbers/biomass to the appropriate area, distribution maps from the SCANS project have been used.

Future scenarios may involve different assumptions about the allocation of animals to the Southern North Sea, and about future levels of gillnetting effort.

## Methods

- Harbour porpoise summer distribution and abundance in the North Sea (NS) were estimated from the SCANS1 and SCANS2 surveys (1994 and 2005). These estimates do not include calves i.e. they represent numbers of 1+ animals.
- To interpolate between these surveys and to extrapolate the population forward in time, a simple production-type model could have been used. However, the age structure of the population may be important not only in estimating biomass, but also in reflecting the time-lags likely to be important in a population of long-lived animals that reproduce at a relatively slow rate (relative to teleost fish).
- In order to interpolate/extrapolate the NS population, to include calves, and estimate changes in overall biomass, a simulation has been coded in R based on the age-structured population model developed by Winship [1]. This model was originally fitted using Bayesian methods to the 2 SCANS estimates, and also made use of substantial prior information based on life-history data from stranded and bycaught animals. The model is initialised with an age structure based on that estimated by Winship during the original model-fitting.
- To convert from numbers-at-age to biomass, a Gompertz growth model was fitted to data described by Van Utrecht [2] using a non-linear least squares routine in order to estimate mass-at-age for both male and female porpoises (females are larger in this species). The age-to-mass calculation is carried out within the simulation so that time series of numbers (calves and 1+ animals) and also of total biomass are produced.
- Bycatch in gillnet fisheries in UK and Danish waters was included in the original population model. The impact of bycatch acts like a fishery, removing individuals from the population. Bycatch effort data available at the time of the modelling exercise were rather complicated.
  - 10 different gillnet fisheries were identified in UK and Danish waters: these were the main source of bycatch for porpoises and other fisheries had relatively little impact.
  - Bycatch rates were recorded as number of bycaught porpoises per unit effort in each fishery.
  - Effort was recorded in two different forms: for UK waters, the number of hauls was used. For Danish waters, catch of target species of the fishery was used. The modelling was possible using this method because bycatch rates could be associated with these measures of effort for each fishery, but the effort data are not easily comparable with one another or with other data.
  - However, extrapolation should be possible. “Inside” the simulation there are 10 fisheries, each with associated effort for each year. If we assume that the distribution of effort between these static fisheries remains fixed, then it is possible to predict the impact of “general” changes in effort in gillnetting. For example, if gillnetting effort in general doubles from 2005 to 2010, a factor 2 can be applied in the simulation to the effort invested in each of the 10 fisheries. This should act to simulate overall changes in static fishing effort and their effects on harbour porpoises. This method is implemented in the R simulation.
- Spatial distribution. Porpoise distribution has changed substantially between the 2 SCANS surveys [3]. The population model predicts number and biomass for the whole North Sea area, but estimates are needed for the southern area. Therefore, we allocate numbers/biomass based on the distribution maps (density surfaces or layers) predicted from the SCANS survey data. Based on these maps, abundance is summed over the grid cells lying in ICES Areas IVb and IVc, and compared with total North Sea abundance. The corresponding proportions of the total population are then allocated to the southern area in 1994 and 2005.
- To estimate the proportion that should be allocated to the south area in other years, between 1994 and 2005 and in future years, a simple linear trend was fitted based on those two years. This was then used to predict changes in the proportion in the south. The linear function was truncated at 1 and 0 so that no negative predictions could be produced and so that the population in the south could not exceed the total population. This is a crude model (a logistic curve might be more aesthetically pleasing) but the trend is relatively gradual so it may suffice.

## Running the model to generate predictions

The R code needed to run a simulation, along with the relevant data e.g. the parameters of the growth model, historical levels of gillnetting effort etc., can be retrieved from S. Smout upon request. The user can decide how many years into the future the simulation will run, what levels of gillnetting effort will be applied (relative to those in 2005). The user can choose either to keep the 2005 spatial distribution of porpoises in the North Sea or else to continue the ‘southward drift’ of the population using a simple linear trend. Therefore the user can apply different scenarios of gillnetting effort and spatial change in order to explore the overall impact on the southern part of the harbour porpoise population in the North Sea.

## Caveats

1. The simulation represents one of several different models that Winship fitted to the SCANS data. It was chosen because density dependence is included and it seems important to include this possibility when dealing with ‘future scenarios’. However, if substantial increases in population size are predicted, these should be regarded with some scepticism. The original model was fitted using data from years when the harbour porpoise population was pretty steady around 250000 in the North Sea. Estimates of density dependence were therefore difficult and uncertain, and the value assumed in the simulation is one of many possible ones.
2. Allocation of gillnetting effort between UK and Denmark, and between gears/target species, might change. Bycatch rates do vary between gears so this might be problematical.
3. The ‘linear model’ used to predict re-distribution is very naïve. Unfortunately we do not yet know what is driving the re-distribution of porpoises (and perhaps seals) towards the southern part of the North Sea. It is therefore difficult to be predictive about it. It seems likely that this is resource-based and would be very interesting to investigate further.

## References

1. Winship A. Estimating the impact of bycatch and calculating bycatch limits to achieve conservation objectives as applied to harbour porpoise in the North Sea: University of St Andrews; 2009.

2. Van Utrecht W. Age and growth in Phocoena phocoena Linnaeus, 1758. Cetacea, Odontoceti from the North Sea Bijdr Dierk. 1978;48:16-28.

3. Hammond PS, Macleod K, Berggren P, Borchers DL, Burt L, Cañadas A, et al. Cetacean abundance and distribution in European Atlantic shelf waters to inform conservation and management. Biological Conservation. 2013;164:107-22.
